# Stochastic Finite Element Analysis Framework for Modelling Electrical Properties of Particle-Modified Polymer Composites

**DOI:** 10.3390/nano10091754

**Published:** 2020-09-05

**Authors:** Hamidreza Ahmadi Moghaddam, Pierre Mertiny

**Affiliations:** Department of Mechanical Engineering, University of Alberta, Edmonton, AB T6G 1H9, Canada; ahmadimo@ualberta.ca

**Keywords:** stochastic finite element analysis, Monte Carlo simulation, particulate polymer composites, electrical conductivity, percolation threshold, piezoresistivity, temperature effects

## Abstract

Properties such as low specific gravity and cost make polymers attractive for many engineering applications, yet their mechanical, thermal, and electrical properties are typically inferior compared to other engineering materials. Material designers have been seeking to improve polymer properties, which may be achieved by adding suitable particulate fillers. However, the design process is challenging due to countless permutations of available filler materials, different morphologies, filler loadings and fabrication routes. Designing materials solely through experimentation is ineffective given the considerable time and cost associated with such campaigns. Analytical models, on the other hand, typically lack detail, accuracy and versatility. Increasingly powerful numerical techniques are a promising route to alleviate these shortcomings. A stochastic finite element analysis method for predicting the properties of filler-modified polymers is herein presented with a focus on electrical properties, i.e., conductivity, percolation, and piezoresistivity behavior of composites with randomly distributed and dispersed filler particles. The effect of temperature was also explored. While the modeling framework enables prediction of the properties for a variety of filler morphologies, the present study considers spherical particles for the case of nano-silver modified epoxy polymer. Predicted properties were contrasted with data available in the technical literature to demonstrate the viability of the developed modeling approach.

## 1. Introduction

Since the middle of the 20th century, polymers have seen rapid deployment in consumer products and industrial applications. Concurrently, researchers have sought to improve polymer mechanical, thermal and electrical properties by adding appropriate fillers [1,2,3,4]. Carbon black (CB) [5,6], carbon nanotubes (CNT) [7,8,9,10] and nano-silver particles [11,12,13,14,15] are some common fillers used for enhancing mechanical, thermal and electrical properties of particulate polymer composites. Industrial applications for such materials include high-voltage and temperature devices, heaters [16,17,18] and electromagnetic interference (EMI) shielding [19,20,21]. The vast diversity of particle materials and morphologies (shape, dimensions, size distribution) poses significant challenges for material designers seeking to effectively develop multifunctional particulate polymer composites that meet desired properties. Analytical and experimental methods are available to explore the material design space. However, analytical methods typically have limited detailedness, accuracy and versatility, while experimental methods are associated with substantial time and cost, making them less attractive. Therefore, new methods for predicting the properties of filler-modified polymers are sought [22,23,24,25,26,27,28,29].

Numerical techniques, especially finite element analysis (FEA), have become popular tools for predicting mechanical [30,31,32,33] and thermal [34,35,36] properties of particulate polymer composites using a representative volume element (RVE) concept. Also, several studies explored the electrical properties of particulate composites using numerical approaches. Pike et al. [37] are pioneers, studying electrical properties such as the percolation threshold, i.e., the minimum amount of filler required for establishing a transfer of electrical charge, in polymer composites using two-dimensional numerical models involving Monte Carlo (MC) simulation. Kirkpatrick [38] and Behnam and Ural [39] also developed two-dimensional numerical models that enabled the prediction of electrical properties of randomly oriented and dispersed CNT in conjunction with an MC approach. Various models have been proposed based on resistor networks to facilitate the prediction of the electrical properties of particulate polymer composites [40,41,42,43,44,45,46,47,48].

The transfer of electrical charge in polymer composites is largely controlled by the quantum mechanical phenomenon of “electron tunneling” [49,50,51,52,53], that is, the transfer of electron electrical charge may occur from one particle to another through an insulator barrier if the distance between the particles is less than an explicit value. This effect brings forth a nonlinear current-voltage relation between two particles. Given a sufficiently high particle concentration and suitable particle dispersion, electrical paths in the form of a continuous conducting structure or network allow electrons and thus electrical current to flow through the material [54]. The percolation model described in Reference [55] considers two types of electrical barriers which are mimicked by electrical resistors, i.e., a tunneling resistance and a contact resistance; the latter relates to particles in direct mechanical contact. The combination of these resistances again gives rise to a nonlinear current-voltage behavior. Notably, it was observed that electrical conduction in particulate polymer composites is affected by temperature [56,57], where an increase in temperature led to an increase in electrical conductivity.

In the context of FEA, the system of equations that represents electrical conductivity, i.e., Ohm’s law, can be written for a linear electrical element as

(1) $$I_{i}^{e}\;=\;\frac{1}{R^{e}}\left(V_{i}-V_{j}\right)$$

where *V_i_* and *V_j_* are the electrical potential (voltage) at nodes *i* and *j*, respectively, *I* and *R* are correspondingly the current and resistance at element *e*. This expression can be written in matrix form as follows:

(2) $$\left\{\begin{array}{c} I_{i}^{e}\\ I_{j}^{e} \end{array}\right\}\;=\;\left[K_{ij}^{e}\right]\left\{\begin{array}{c} V_{i}\\ V_{j} \end{array}\right\}$$

where $K_{ij}^{e}$ is known as the electrical stiffness matrix, which is defined as

(3) $$\left[K_{ij}^{e}\right]\;=\;\frac{1}{R^{e}}\left[\begin{array}{cc} 1 & -1\\ -1 & 1 \end{array}\right]$$

The effective electrical properties, i.e., the effective electrical conductivity of an RVE (representing e.g., a particulate polymer composite), can be calculated as follows [44,45]:

(4) $$EC_{i}\;=\;\frac{I_{i}\times D}{\left(V_{L}\;-\;V_{R}\right)}$$

(5) $$EC_{eff}\;=\;\overset{n}{\underset{i\;=\;1}{\sum }}EC_{i}$$

where *EC_i_* and *I_i_* are correspondingly the electrical conductivity, in units of Siemens per meter (S/m), and electrical current density (units A/m^2^), at the *i*-th node located on the face of an RVE exposed to an electrical charge. *D* is the RVE characteristic length. *V*_L_ and *V*_R_ are voltages that are applied to opposing RVE faces, i.e., a ‘back’ and ‘front’ side. *EC*_eff_ is the effective electrical conductivity, with *n* being the number of nodes located on each of the RVE faces exposed to an electrical charge.

The distribution of particles in polymer composites, and thus its electrical properties, are statistical in nature. Hence, in this paper, a stochastic FEA (SFEA) framework was employed that enables prediction of the effective electrical conductivity and percolation threshold of particulate polymer composites. Interested readers are referred to Reference [58] for detailed information on the SFEA framework concept, including a consideration associated with MC simulation and random number generation for creating true randomness.

Applying mechanical strain to a polymer modified with a conductive filler such as nano-silver particles may result in the phenomenon known as piezoresistivity, that is, resistivity changes occur as the material is subjected to mechanical strain. This effect is the result of changing distances between conductive particles, as well as changes in particle orientation for cases when orientation matters, i.e., non-spherical particles (e.g., cylindrical, ellipsoidal, and disk-shaped particles). Materials exhibiting piezoresistivity may be good candidates for making sensors that provide deformation-based measurements.

Several numerical and analytical methods have been developed to investigate the piezoresistivity of particulate polymer composites [59,60,61,62,63]. In many of these studies, a resistor network was created to represent the particles and their electrical interaction; the polymer matrix was typically not explicitly modeled as a continuum. Such a modeling approach, while expedient, fails to capture mechanical interactions of the composite constituents, such as the deformation of particles due to arising stress/strain in the composite, and hence, the final model predictions may be compromised. In contrast, the SFEA framework employed in the presented study includes both the matrix material and embedded particles in order to predict piezoresistivity. It is postulated that accurate results can thus be achieved since this approach enables the calculation of particle locations, orientations, and deformations precisely as a result of not only considering the global but also the local mechanical strain. Moreover, the effect of material parameters Poisson’s ratio and Young’s modulus on piezoresistivity can be investigated.

Employing the SFEA framework, filler-modified polymers were herein modeled, and their electrical properties predicted (i.e., conductivity, percolation and piezoresistivity), including the effect of temperature. The composites comprised randomly distributed and dispersed filler particles. Spherical nano-silver particles embedded in epoxy polymer were considered in this study. Modeling results were compared with values from the technical literature in order to demonstrate the viability of the developed modeling approach.

## 2. Overview of SFEA Framework

**Figure 1:**
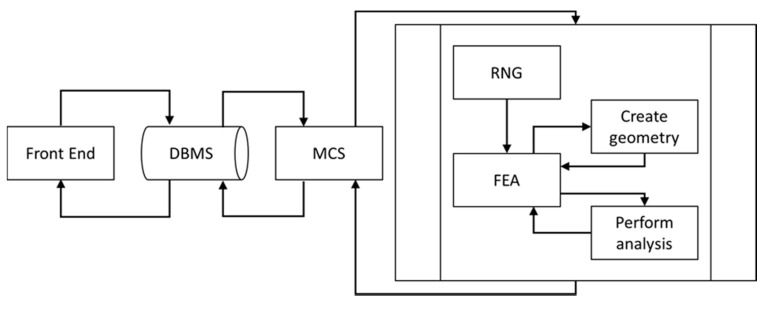
Schematic of the SFEA framework [58].

In order to predict the effective electrical conductivity and percolation threshold of particulate polymer composite, an SFEA framework was created using multiple programming languages. A schematic of the framework is depicted in Figure 1.

**Figure 2:**
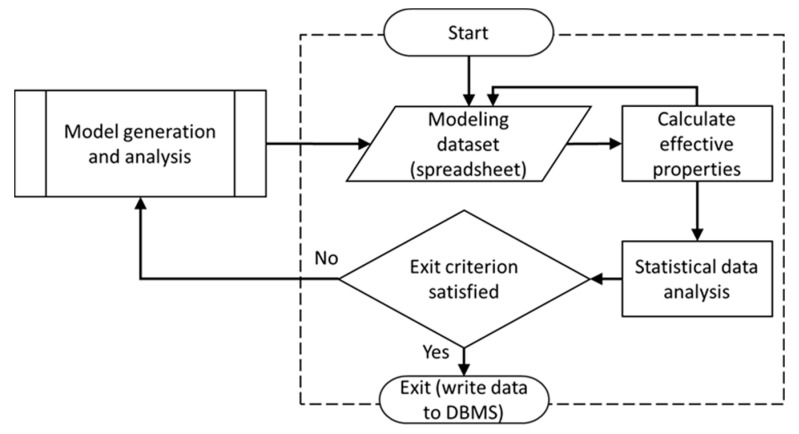
A schematic illustrating the algorithm for the Monte Carlo Simulation (MCS) module [58].

Visual Basic for Applications (VBA; Microsoft, Redmond, WA, USA) programming language was used to create a domain that connects the various modules developed for the framework. The user interacts with the framework via the “Front End”, which was written in VBA programming language. The Front End enables the capture of information required for the analysis, including RVE size, particle size distribution, electrical properties (e.g., the electrical resistivity of polymer matrix as well as filler, electrical conductance between polymer matrix and particles, the tunneling distance and electrical conductance between particles, electrical boundary conditions), and finally parameters defining the mesh for the FEA model. Data captured by the Front End are stored in tabulated format within a database. An Open Database Connectivity concept was used to enable accessing the Database Management System (DBMS) module. This method facilitates access to the database at any time during the numerical analysis. All information saved in the DBMS module is transferred to the Monte Carlo Simulation (MCS) module, which is the core of the SFEA framework. The MCS module was developed in tabulated format using VBA programming language, which enables storing input parameters as well as saving results calculated by SFEA framework as illustrated in Figure 2. The subprocess shown in Figure 1 is iterated as part of the MCS module in order to calculate the effective electrical conductivity and probability of passing the electrical current from one side of an RVE to the other in order to identify the percolation threshold as the volume fraction is increased. Once a user-defined terminating number of iterations is reached, or the standard deviation of the dataset is below a threshold set by the user prior to starting the SFEA framework execution, the MCS module stops iterating, and calculated results are transferred and stored in the database, which can be accessed by the user from the Front End. In typical fashion, increasing the number of iterations will increase the accuracy of predicting the effective electrical conductivity as well as the percolation threshold for the simulated material system. 

**Figure 3:**
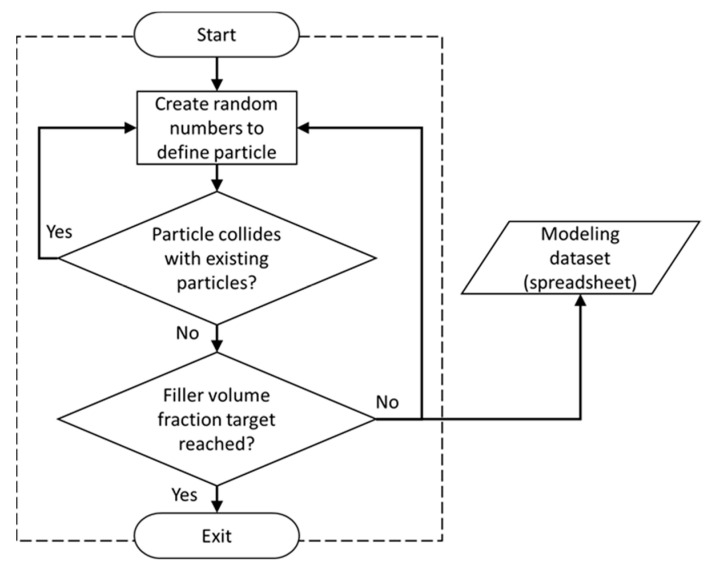
A schematic illustrating the Random Number Generator (RNG) module [58].

The MC subprocess, as illustrated in Figure 1, starts with the Random Number Generator (RNG) module, for which a schematic is shown in Figure 3. This module facilitates the process of generating random numbers required for creating the particulate polymer composite morphology, e.g., particles’ coordinates and size distribution. Interested readers are referred to Reference [58] for additional information on how the RNG module creates random numbers that conform to a given particle size distribution. The RNG module was developed in the general mathematical programming environment MATLAB (MathWorks, Natick, MA, USA) with the goal of creating true randomness in the SFEA framework.

Since in an actual material, particles do not intersect with each other, i.e., they cannot occupy the same space, the RNG module performs a collision detection for the particles contained within the RVE. If a newly added particle intersects with either the RVE surface or other particles already within the RVE, the particle is rejected, and a new particle is created instead. This process continues until the volume fraction defined by the user in the Front End is satisfied. The RNG module stores the data in tabulate format within a database which is accessed through the FEA module for creating the finite element model.

**Figure 4:**
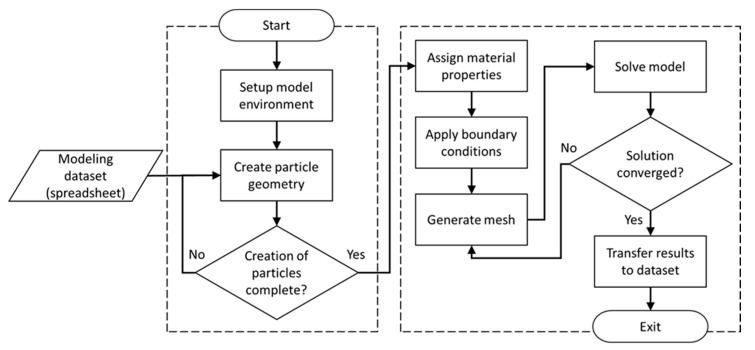
Schematic illustrating the algorithm for the FEA module [58].

The commercial FEA software package ANSYS Workbench (Version 19, ANSYS Inc., Canonsburg, PA, USA) was employed for creating the material model. IronPython programming language was used for developing a customized FEA module as shown in Figure 4. Since in ANSYS Workbench the model generation environment (ANSYS DesignModeler) is separate from the FEA solution environment (ANSYS Mechanical), two different customized modules were developed using JavaScript programing language. The setup enables automating of the process of reading random numbers from the database, creating the particulate polymer composite geometry (i.e., the RVE in DesignModeler), assigning electrical properties to the polymer matrix and the particles, defining contact between particles and the polymer matrix as well as contact between particles (i.e., ‘contact’ implies the tunneling phenomenon), forming electrical boundary conditions, and setting parameters required for mesh generation. Results in terms of electrical conductivity calculated for each iteration are transferred to the MCS module for storage in tabulated format for further statistical analyses.

## 3. Steady-State Electric Conduction Numerical Model

### 3.1. Electrical Conduction and Percolation Threshold Modeling

Steady-state electric conduction numerical modeling was performed using the SFEA framework for predicting the effective electrical conductivity and electrical percolation threshold of particulate polymer composites. In the present paper, material systems with spherical-shape particles were modeled, and it was decided to predict the electrical properties of silver nano-particles embedded in an epoxy polymer matrix. The electrical properties of particles and matrix as shown in Table 1 were considered for the model. Parameters determining particle sizes, for use with the RNG module, were adjusted so that the filler conforms to a distribution with average particle diameters of 3 nm, 5 nm and 7 nm with a size variation of ±5 percent from the mean. These sizes were adopted from TEM images presented in Reference [14], where nano-silver particles were reported to aggregate forming clusters. The present modeling approach could thus serve to explore the properties of nano-silver clusters or an assumed macro nanocomposite with well-dispersed and distributed nano-particles. In terms of RVE size, a desirable dimension would ensure the true randomness of the model. Hence, as suggested in Reference [61], the RVE size was set to ten times greater than the particle dimensions, which was found to be large enough to satisfy randomness in the model.

**Figure 5:**
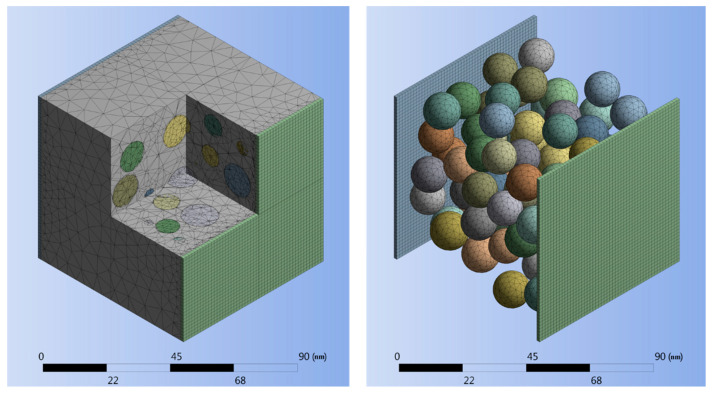
A cut-away view of the finite element mesh for RVE and electrical charge plates (**left**) and an isolated view of electrical charge plates and particles (**right**) for the 0.3 filler volume fraction.

Three-dimensional twenty-node electric solid elements (SOLID231) were used for generating the mesh for two electric charge plates placed at the back and the front-side of the RVE, as illustrated in Figure 5. The chosen element, which is based on an electric scalar potential formulation, has only one degree of freedom (voltage) at each node and can be used for modelling irregular shapes without losing accuracy. Three-dimensional ten-node quadratic tetrahedral electric solid elements (SOLID232), carrying only one degree of freedom (voltage) were used for modelling the polymer matrix as well as the filler. Nodes located in the plane of contact between the polymer matrix and the electrical charge plates were merged to avoid any discontinuity in the model and increase result accuracy.

**Figure 6:**
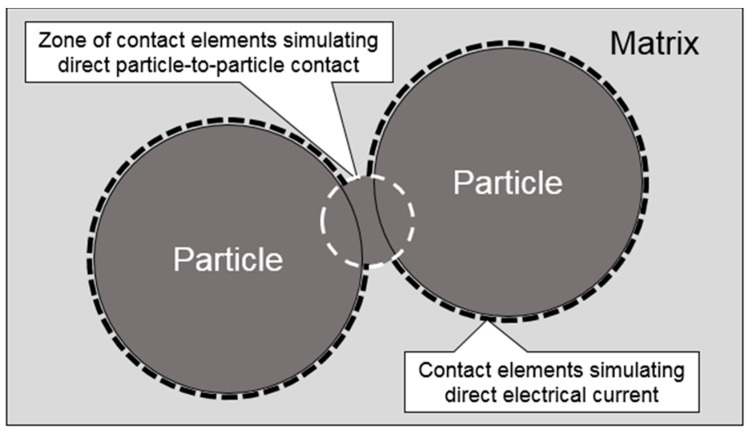
A schematic depicting the modeling approach for direct particle-to-particle electrical contact.

Two forms of contact, i.e., particle-to-matrix and particle-to-particle, were implemented to essentially establish a numerical resistor network. The modeling concept enabling a direct particle-to-particle electrical current is schematically depicted in Figure 6. Interested readers are referred to Reference [58] for further information on the contact element zone simulating direct particle-to-particle contact (in the context of heat transfer). Three-dimensional six-node quadratic surface-to-surface structural-thermal-electric coupled field elements (CONTA174 and TARGE170) were used for modelling electric current conduction between the RVE constituents. Since structural and thermal aspects were not the focus of the present analysis, KEYOPT (1) was used to set the required degree of freedom for modelling electric contact. The surface electric interaction between the polymer matrix and particles was defined employing the concept of ‘electric contact conductance’ (ECC) per unit area as described by Equation (6).

(6) $$J\;=\;ECC\left(V_{t}-V_{C}\right)$$

where *J* and *ECC* are the current density and electric contact conductance for an electric potential (voltage), respectively; *V*_t_ and *V*_C_ are correspondingly the voltages at the target and contact surfaces. While the ECC can be a function of temperature and pressure existing at the contact, in this study, temperature and pressure effects were neglected. A small ECC of 10^−4^ S/m^2^ was used for defining the contact from the polymer matrix to particles and electric charge plates. Due to this contact setting, the polymer matrix has only a minimal contribution to the effective electrical conductivity in the resistor network, which is akin to other works employing a resistor network method [40,41,42,43,44,45,46,47,48]. However, since the present authors seek to also explore the effects of applied mechanical strain and temperature change on the effective electrical conductivity, representing the matrix in the numerical model is crucial for enabling sequential multiphysics simulations.

**Figure 7:**
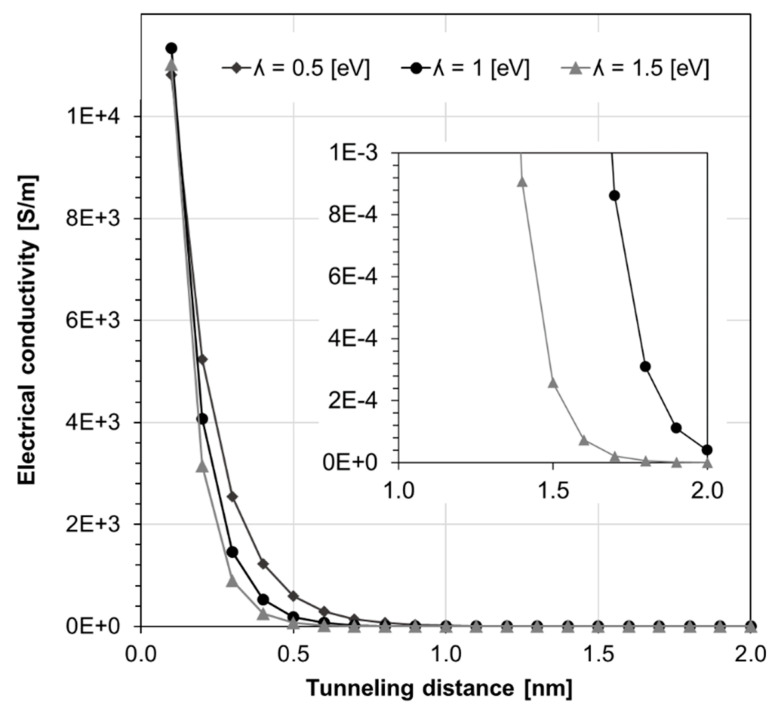
Electrical conductivity versus tunneling distance for different polymer barrier heights *λ*, based on Equation (7).

As mentioned previously, the electron tunneling effect dominates the electrical conductivity of particulate polymer composites, and hence, modeling this effect with the SFEA framework is a key aspect for predicting the effective electrical conductivity and percolation behavior. A script was written in JavaScript programming language that automatically measures the distance between particles within the RVE, as well as the distance between particles and electrical charge plates, for defining another type of contact in ANSYS Mechanical. If the measured distance was less than an explicit threshold, i.e., the tunneling distance, a contact was defined that permits a transfer of electrical charge. The minimum distance required for transferring a charge can be measured experimentally [11,12]. The ‘tunneling’ contact was created using three-dimensional six-node quadratic surface-to-surface structural-thermal-electric coupled field elements (CONTA174 and TARGE170), which allow ‘direct electrical conduction’ (DEC) between particles within the RVE when the distance between particles and the distance between particles and an electric charge plate is less than the tunneling distance. The ECC value was approximated based on Equation (7) [64].

(7) $$\rho_{\mathrm{tunl}}\;=\;\frac{h^{2}}{e^{2}\sqrt{2m\lambda }}\exp\left(\frac{4\pi d}{h}\sqrt{2m\lambda }\right)$$

where *m* and *h* are the electron mass and Planck’s constant, respectively; *λ* and *e* are correspondingly the polymer barrier height and the quantum of electricity; and *d* is the parameter defining the tunneling distance. Upon the material system reaching percolation, Equation (7) affects the material electrical resistivity, imposing a nonlinear behavior between tunneling distance and electrical resistivity. As suggested in Reference [11], the barrier height can vary from 1 eV to 4 eV. In this study, *λ* was chosen as 1.5 eV. The technical literature describes a range of experimentally measured tunneling distances [11,12,13] varying from 0.5 to 5 nm. For the present analysis, a tunneling distance of 1 nm and 1.5 nm was set for assessing percolation behavior and effective electrical conductivity. As shown in Figure 7, electrical conductivity diminishes for tunneling distances greater than 1.5 nm for the considered barrier height value of 1.5 eV.

**Figure 8:**
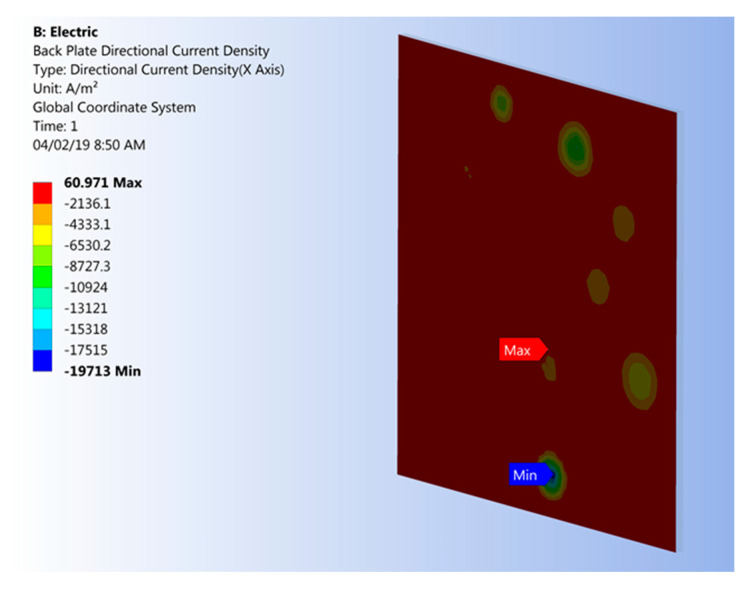
Example of simulated electrical current density on an electrical charge plate after achieving percolation conditions.

In terms of RVE boundary conditions, a low electrical potential of 0.2 V was applied between the electric charge plates. In the case of electrical percolation, this boundary condition produces direct current (DC) flowing between the electrical charge plates. It is a necessary condition that the average current density on all nodes located on the sides of the RVE with an electrical charge plate, i.e., the total current entering and exiting the RVE, is equal. Figure 8 depicts an example of an electrical current density distribution that was simulated after a percolation condition was achieved. It is interesting to note that the minimum number of electrical paths required for achieving percolation is one. Therefore, in models with low filler loading, a large portion of particles may not influence the electrical properties of the polymer composite. Particles located in the electrical path are known as “backbone” particles [38] with non-zero current [46].

### 3.2. Electrical Piezoresistivity Modeling

The developed SFEA framework was also employed for exploring electrical piezoresistivity effects of particulate polymer composites. For this part of the study, the average particle diameter was set to 3 nm with a size variation of ±5 percent from the mean. An RVE size of 30 nm was found to be sufficient to achieve random material systems. For the deformation-based analyses, mechanical properties for the filler and matrix were used as shown in Table 2.

A static structural numerical model was created using a three-dimensional ten-node quadratic tetrahedral structural solid elements (SOLID187) for generating the finite element mesh for particles and the matrix, as depicted in Figure 5. Three-dimensional eight-node surface-to-surface contact elements (CONTA174 and TARGE170) were used for defining the contact between particles and the matrix, restraining any relative displacement between a particle and the surrounding matrix.

The following boundary conditions were applied to the static structural numerical model to simulate the displacement and deformation of particles within the RVE. A Cartesian displacement boundary condition was applied to the RVE that restrained nodes located on one face from moving perpendicular to the face while permitting displacements in the transverse direction. Another uniform Cartesian displacement boundary condition was applied to the opposing face that forced all the nodes located on this surface to displace by an explicit value in the direction perpendicular to the surface, again with the freedom to displace laterally. To restrain the RVE from rigid body motion, a Cartesian zero displacement boundary condition was applied to one of the RVE’s corners.

The above boundary conditions enabled applying mechanical strain to the material system and predicting changes in composite morphology. A script was developed using JavaScript programming language that facilitates extracting the geometry data of the deformed body (i.e., particle sizes and location coordinates) and the saving of this information in tabulated format. This data was used for generating a post-deformation steady-state electric conduction numerical model (as explained in the previous section) in order to calculate the effective electrical conductivity of the deformed material system.

### 3.3. Thermal-Electrical Numerical Model

The developed SFEA framework was further used to explore the effects of temperature on the effective electrical conductivity of a particulate polymer composite. The same material system was herein used as described in the section on piezoresistivity modeling above. In addition to mechanical properties, the coefficients of thermal expansion, as shown in Table 3, were used for the nano-silver and epoxy material.

A sequential structural-thermal numerical model was created for calculating the effective electrical conductivity of particulate polymer composites subjected to temperature change. The same element types, as well as mesh properties described in the previous section, were employed for this modeling approach since the utilized elements possess the degrees of freedom required for considering temperature in the numerical model.

Mechanical boundary conditions were set akin to the piezoresistivity model. In addition, a body temperature was applied to the RVE, enabling the simulation of a temperature change from ambient conditions, i.e., an initial temperature of 22 °C, to an elevated temperature. The applied mechanical-thermal boundary conditions thus impose the thermal expansion of both the particles and polymer matrix, and in consequence, changes in the location and size of particles, which in turn may affect the material electrical properties. The JavaScript program described in the previous section was again used for extracting and saving particle locations and sizes for performing a steady-state electric conduction numerical model for calculating the effective electrical conductivity following a temperature change.

## 4. Results and Discussion

### 4.1. Effective Electrical Conductivity and Percolation Behavior

**Figure 9:**
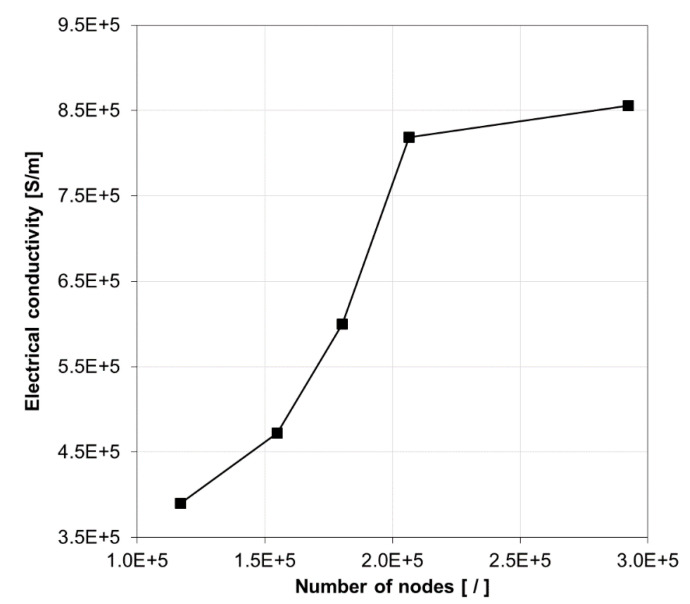
Electrical conductivity for different model mesh refinements for 21 vol% filler loading.

The effective electrical conductivity for an epoxy nanocomposite with nano-silver particles was computed using the aforementioned properties for filler volume fractions ranging from 3 vol% to 30 vol% with an interval of 3 vol%. The first step of the analysis was performing a convergence study where the effective electrical conductivity of the composite was determined for a few different levels of mesh refinement. Results shown in Figure 9 are for the case of 21 vol% filler loading and particle size and tunneling distance 3 nm and 1.5 nm, respectively. It was observed that the effective electrical conductivity changed by less than 5 percent when increasing the number of nodes from approximately 210,000 to 290,000, and hence, the mesh refinement corresponding to 210,000 nodes was deemed to be sufficiently fine for being employed in the SFEA framework for all volume fractions. Due to the stochastic nature of the chosen modeling approach, numerous model runs are needed for calculating the effective properties; therefore, minimizing the number of nodes in the model is critical for maintaining acceptable computing times required for a problem solution. Note that the spatial particle distribution generated by the SFEA framework was already investigated in Reference [58]. Interested readers are referred to this publication for a discussion on the performance of this modeling framework to generate randomly distributed particles inside the RVE.

**Figure 10:**
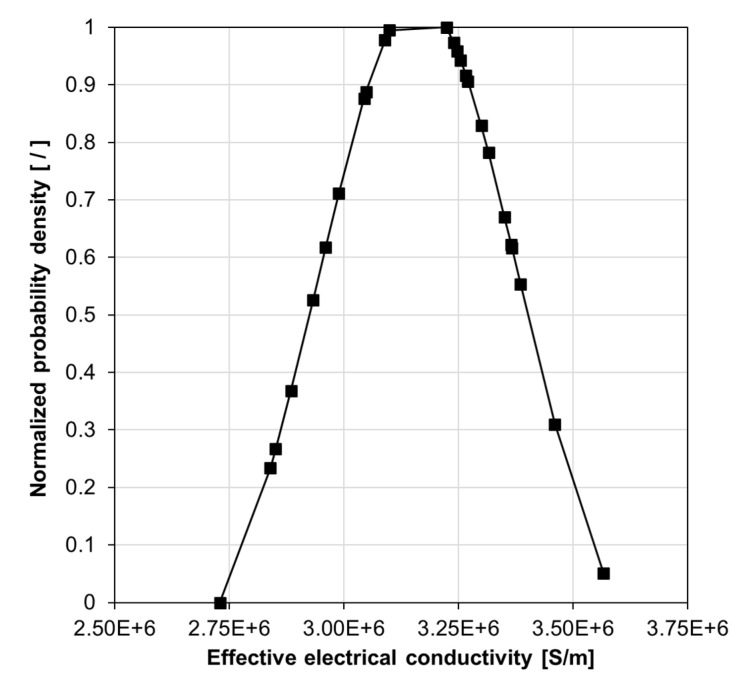
A normalized probability density graph for effective electrical conductivity data of a silver/epoxy nanocomposite system with a particle size of 3 nm, tunneling distance of 1.5 nm, and 30 vol% filler loading.

As mentioned previously, the RVE effective electrical conductivity is computed and stored in each model iteration. This data is used for statistical analyses, such as calculating the unbiased standard deviation and variance for a dataset reflecting the mean effective electrical conductivity for a given material configuration. Abiding by the MC simulation concept, the mean of the effective electrical conductivity results from a set of model iterations was calculated and taken as the final effective electrical conductivity for a given filler volume fraction. For example, Table 4 shows the mean effective electrical conductivity and statistical analyses performed for a material system with a filler volume fraction of 30%, filler particle size of 3 nm, and tunneling distance of 1.5 nm. Simulation data are further depicted in Figure 10 in the form of a normalized Probability Distribution Function (PDF), which suggests that the results are closely normal distributed. Readers are referred to Reference [58] for a discussion on how data calculated by the SFEA framework conforms to a normal distribution based on statistical analysis results and acceptance criteria such as mean, median, skewness, and kurtosis values. Note that in the present work, being mindful of required computational resources and solutions times, the number of model iterations was limited to 25 for each material configuration.

**Figure 11:**
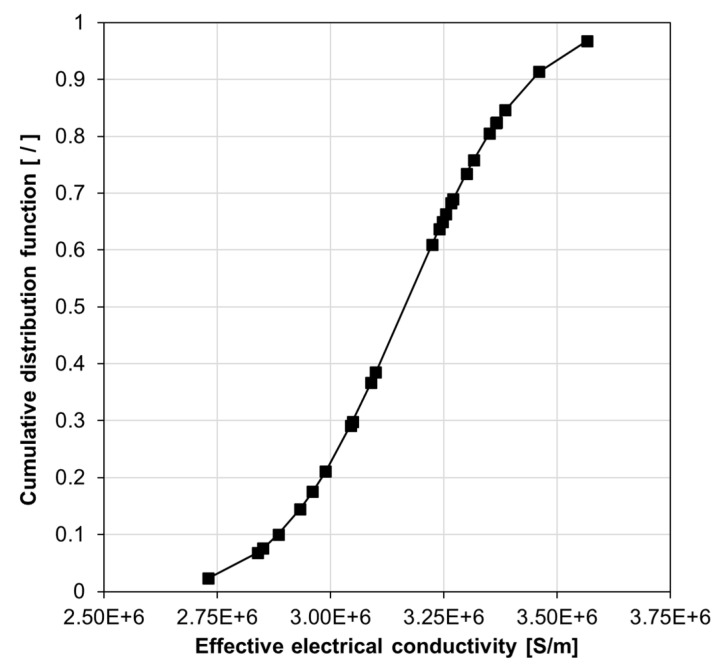
The cumulative distribution function for effective electrical conductivity data of a silver/epoxy nanocomposite system with a particle size of 3 nm, tunneling distance of 1.5 nm, and 30 vol% filler loading.

Simulation results can be considered continuous random variables, and therefore, it is possible to calculate the probability of a specific effective electrical conductivity happening within an explicit interval using Equation (8).

(8) $$P\left(a\leq Χ\leq b\right)\;=\;\int_{a}^{b}f\left(\chi \right)d\chi$$

where *P* is the probability of an effective electrical conductivity occurring within an interval *a* and *b*; *f*(*χ*) and *χ* are the PDF of the data set and a continuous random variable, respectively. Hence, a Cumulative Distribution Function (CDF) can be computed for each of the various filler volume fractions for a specific material system. For example, Figure 11 depicts the CDF graph for the material system corresponding to Figure 10.

**Figure 12:**
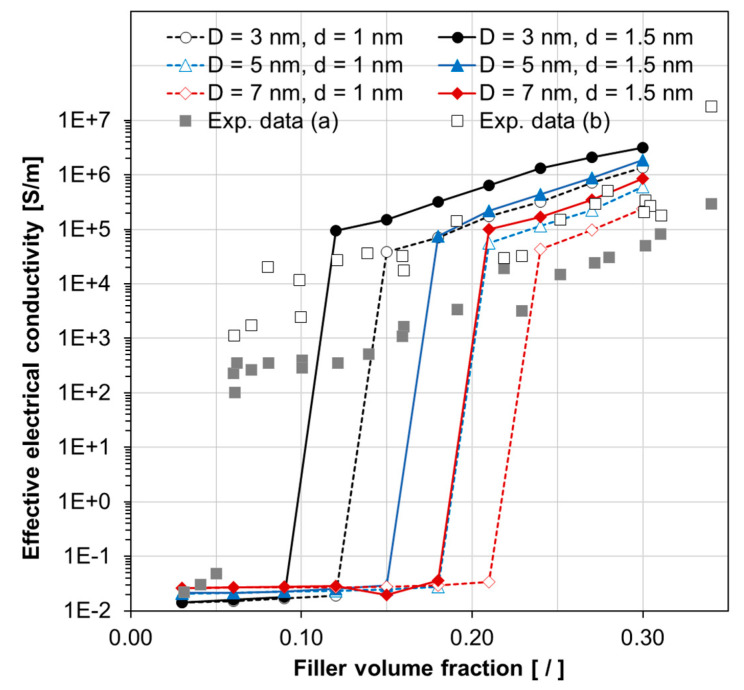
Predicted effective electrical conductivity versus filler volume fraction for silver/epoxy nanocomposites with different particle sizes (*D*) and tunneling distances (*d*). Experimental (Exp.) data (square symbols) are taken from Reference [14], with (**a**) and (**b**) indicating tests before and after thermal treatment, respectively.

The developed SFEA framework was used for predicting effective electrical conductivities of different material systems, i.e., for particle sizes, *D*, of 3 nm, 5 nm and 7 nm and tunneling distances, *d*, of 1 nm and 1.5 nm. The corresponding results are depicted in Figure 12. As mentioned earlier, each predicted data point represents the mean value of 25 model iterations. Figure 12 also shows experimental data, taken from Reference [14], for a specific nano-silver epoxy material before and after thermal treatment. While the experimentally observed percolation threshold was between 5 vol% and 6 vol%, percolation was predicted to occur at higher filler loadings (i.e., >10 vol%) for the simulated material systems. Above percolation, the predicted and experimental data are qualitatively and quantitatively in good agreement, especially for the test data obtained for the nanocomposites after thermal treatment. As mentioned above, a material morphology with clustered silver particles was observed in Reference [14], whereas the present modeling approach generated homogenously distributed and well-dispersed particles, which is likely the cause for the differences in percolation behavior between experiments and modeling results. Nevertheless, despite morphological differences, the SFEA framework was able to simulate material systems that closely mimic actual material behavior.

**Figure 13:**
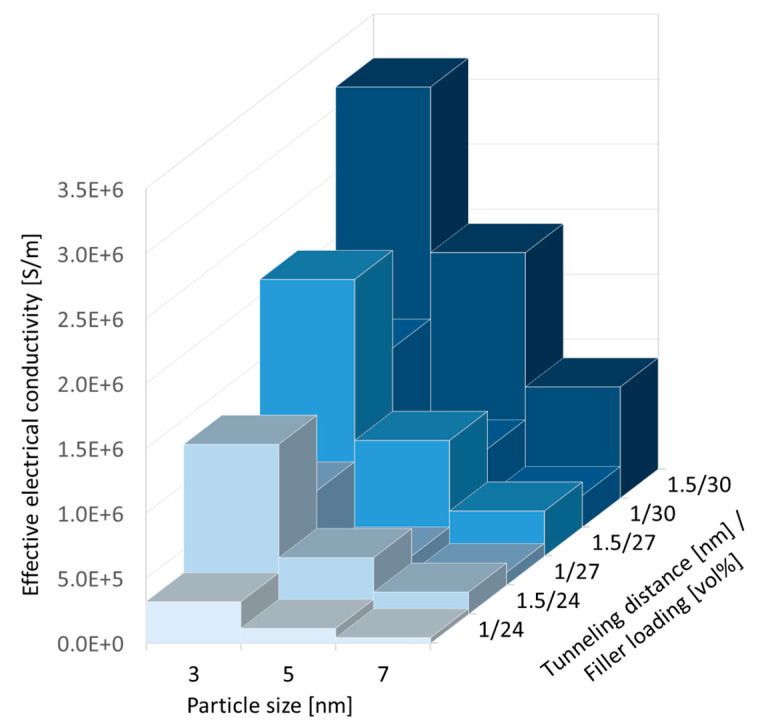
The influence of filler loading, tunneling distance, and particle size on the effective electrical conductivity data for silver/epoxy nanocomposites above percolation.

Effective electrical conductivity data above percolation were plotted in Figure 13 to explore the influence of the key independent modeling parameters (i.e., filler loading, tunneling distance and particle size). From this graph, it can be inferred that, expectedly, filler loading chiefly influences effective electrical conductivity. For the range of considered particle sizes and tunneling distances, both parameters were found to also have significant influence. Given that the tunneling distance is difficult to quantify compared to particle size and filler loading, and considering its impact on modeling outputs, careful consideration should be given when exploring material designs.

**Figure 14:**
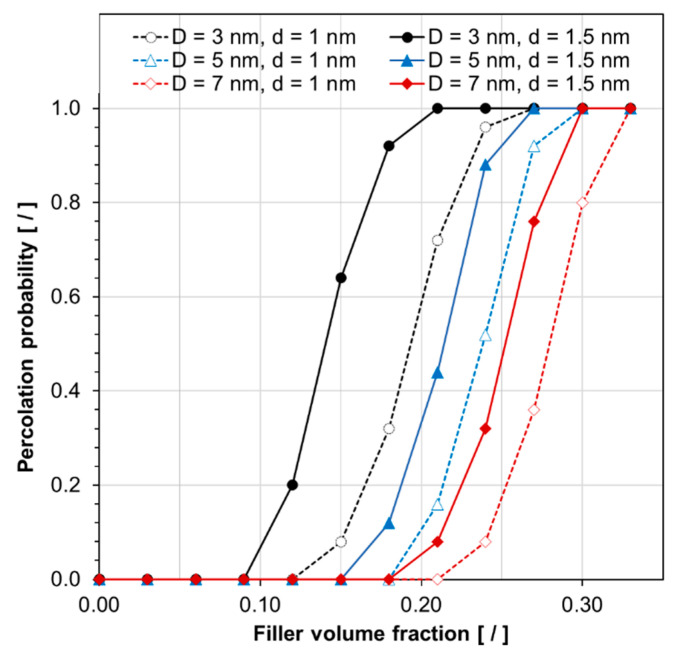
The probability of reaching electrical percolation, for an effective electrical conductivity threshold of 1.0 S/m, versus filler volume fraction for silver/epoxy nanocomposites with different particle sizes (*D*) and tunneling distances (*d*).

**Figure 15:**
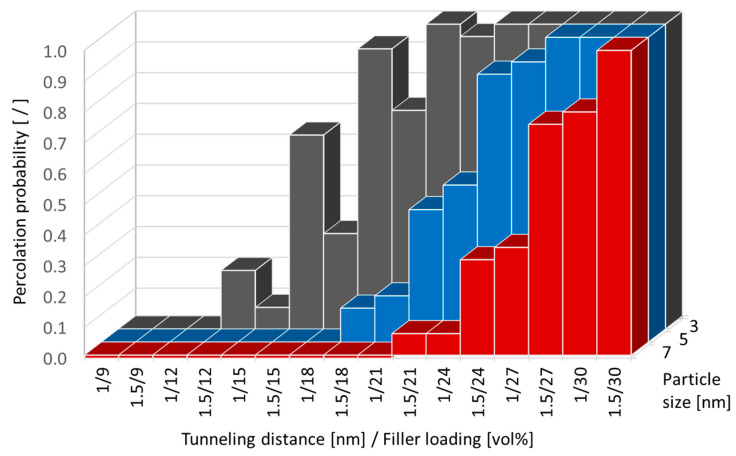
The influence of filler loading, tunneling distance, and particle size on the probability of for a silver/epoxy nanocomposite reaching electrical percolation, for an effective electrical conductivity threshold of 1.0 S/m.

Besides effective electrical conductivity, it is also of interest to assess the influence of the key independent modeling parameters on the percolation behavior. An arbitrary yet sensible effective electrical conductivity value of 1.0 S/m was set as the threshold for deciding that electrical conduction through the RVE is established, i.e., electrical percolation is achieved. Furthermore, the probability of reaching the percolation threshold for any given volume fraction was calculated using Equation (9).

(9) $$P_{\mathrm{th}}\left(E\right)\;=\;\frac{A}{N}$$

where *P*_th_ is the probability of reaching the percolation threshold for a given material system; *A* and *N* specify the number of model iterations that the effective electrical conductivity was above the threshold value and the total number of iterations, respectively. Corresponding results are summarized in Figure 14 and Figure 15. Similar to the electrical conduction behavior, these results indicate the substantial effect that tunneling distance and particle size have on reaching percolation, with particle size being the most significant parameter.

### 4.2. Piezoresistivity Behavior

**Figure 16:**
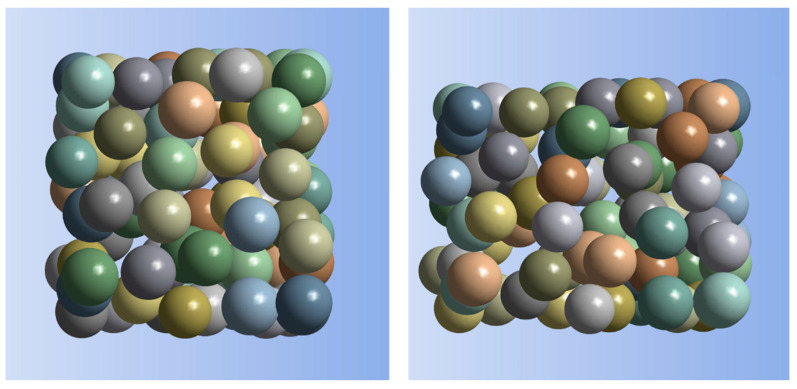
An illustration of the applied tensile strain and Poisson’s effects on filler particle proximity: particles in undeformed RVE (**left**), and particles in deformed RVE (**right**), for 90,000 microstrain applied to the RVE (horizontal), RVE and particle size of 30 nm and 5 nm, respectively, and filler loading of 30 vol%. For clarity, the matrix material is not shown, and deformations and displacements are depicted with a 150× scaling factor.

**Figure 17:**
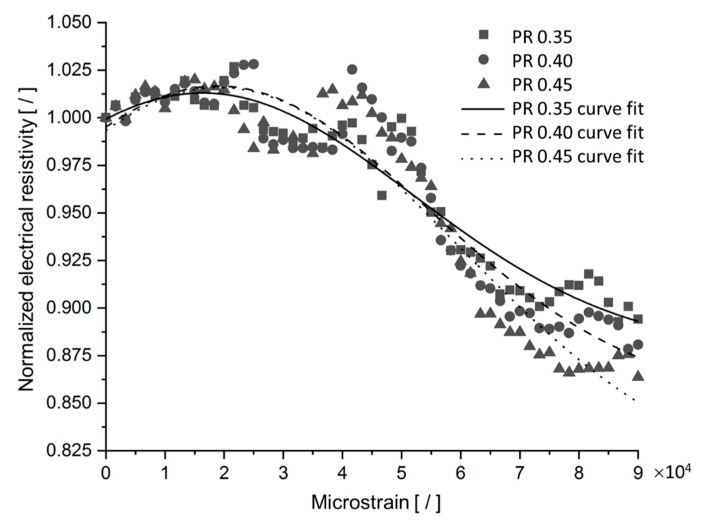
Normalized effective electrical resistivity versus applied mechanical strain for silver/epoxy nanocomposites with a particle size of 3 nm, tunneling distance of 1.5 nm, filler loading of 30 vol%, and polymer matrix Poisson’s ratio (PR) of 0.35, 0.40, and 0.45.

A possible application of silver/epoxy nanocomposites are sensors; for example, for measuring deformation. Hence, in the second part of the present research, the SFEA framework was used to investigate the piezoresistivity behavior of these nanocomposites. The piezoresistive behavior of conductive filler modified polymers can be rather complex. For example, the applied tensile strain does not simply cause the distance between filler particles to increase; while filler proximity increases between some particles, it also decreases simultaneously between other particles due to Poisson’s effects in a continuum material, as illustrated in Figure 16. An analysis was conducted imposing a mechanical strain of up to 90,000 microstrain upon a material system with a particle size of 3 nm, RVE size of 30 nm, tunneling distance of 1.5 nm, and filler loading of 30 vol%. Electrical resistivity data were computed considering three different Poisson’s ratios for the polymer matrix, i.e., 0.35, 0.4, and 0.45. Corresponding results are shown in Figure 17 along with non-linear Gaussian curve fits. Note that each data point in this figure was computed using a single iteration of the SFEA framework. As shown previously, results for a specific material system are subject to considerable stochastic variation, which becomes rather evident in the plotted datasets. Nevertheless, it can be seen that the material systems are sensitive to applied strain in a non-linear fashion, which is in agreement with previous modeling work on other nanocomposites with conductive platelet fillers [59]. After an initial increase in resistivity by approximately 2.5% at about 20,000 microstrain, a decrease in resistivity by roughly 10% over the initial value was predicted at the maximum applied strain. The data show that the matrix Poisson’s ratio has only minor effects over a large portion of the assessed strain range. From the presented results, two shortcomings pertaining to a potential sensor material can be noted. First, the sensor response cannot be linked uniquely to a certain strain value as resistivity initially rises before decreasing at higher strain values. Secondly, the considerable stochastic variation in response behavior between different samples of the same material configuration requires careful sensor calibration.

### 4.3. Temperature Effect on Effective Electrical Conductivity

Assessing the effects of temperature on the effective electrical conductivity is important when considering applications of silver/epoxy nanocomposites. The material response at different temperatures is affected by two physical phenomena: (i) changes in tunneling distance due to thermal expansion effects, and (ii) the effect of temperature on electron activity. With respect to the former phenomenon, a rising temperature changes the material volume, and therefore the distances between particles depending on the thermal-mechanical properties of the matrix and particle filler. The other contributing phenomenon, electron activity changing with temperature, has been described in terms of tunneling current density for intermediate voltages, where *eV* ≤ *λ*, as given by Equation (10) [65].

(10) $$J\left(V,T\right)\;=\;J\left(V,T_{0}\right)\left\{1+\left[\frac{3\times 10^{-9}\times d^{2}T^{2}}{\lambda -\frac{V}{2}}\right]\right\}$$

where *J*, *V,* and *T* are current density, voltage, and temperature in degrees Kelvin, respectively; *T*_0_ represents absolute zero Kelvin (−273.15 °C).

**Figure 18:**
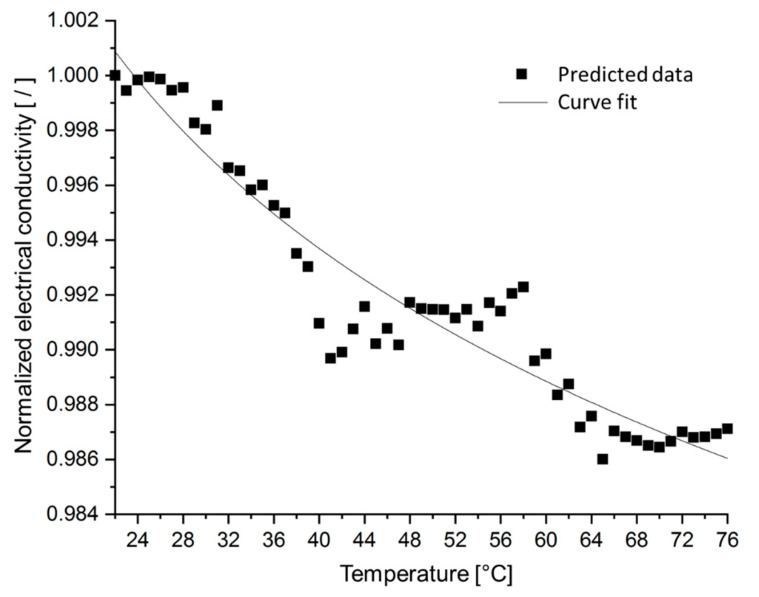
Normalized electrical conductivity, considering only thermal expansion effects, versus temperature for silver/epoxy nanocomposites with a particle size of 3 nm, tunneling distance of 1.5 nm, and filler loading of 30 vol%.

**Figure 19:**
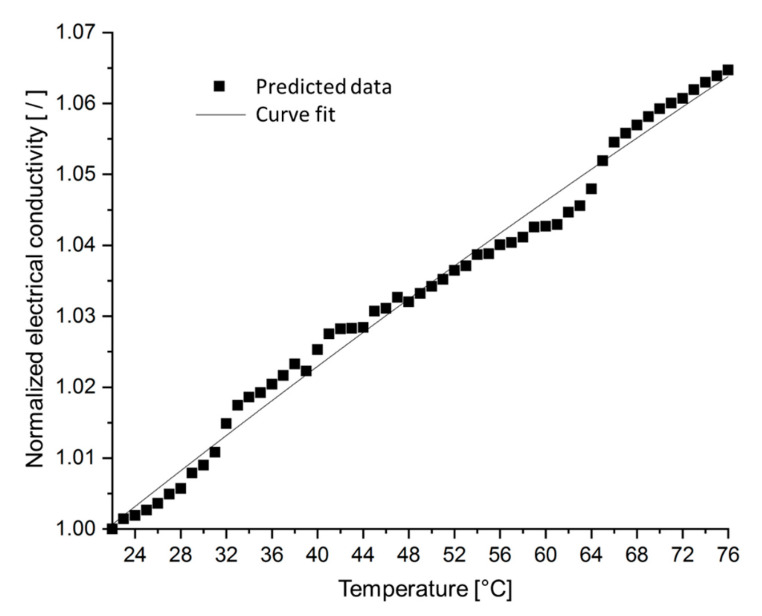
Normalized electrical conductivity, considering thermal expansion and electron activity effects, versus temperature for silver/epoxy nanocomposites with a particle size of 3 nm, tunneling distance of 1.5 nm, and filler loading of 30 vol%.

Since the developed SFEA framework explicitly considers both the matrix and particulate filler, it enables studying the effects that both phenomena have on a nanocomposite’s electrical conductivity. Taking the same material system as in the previous section, a temperature change was applied to the model ranging from ambient 22 °C to 76 °C. Electrical conductivity data were computed, and results are shown in Figure 18 considering only thermal expansion effects, whereas the influence of temperature on electron activity as described by Equation (10) is included in the data shown in Figure 19. As in the previous section, each data point in these figures represents only a single model run in order to demonstrate the extent of stochastic data variation. The data shown in Figure 18 and Figure 19 indicate that thermal expansion effects cause a slight reduction in electrical conductivity over the given temperature range (less than 2%). However, when considering electron activity, the model data exhibits a moderate increase in electrical conductivity (by about 7%). Clearly, both thermally-induced phenomena are counteractive.

## 5. Conclusions

A stochastic finite element analysis framework was developed that enables predicting the electrical conductivity behavior of polymer composites with electrically conductive fillers. The analysis framework establishes a resistor network that encompasses a continuum representation of both the matrix material and filler particles. As such, the modeling approach enables estimating the composite percolation behavior, and provides a means to simulate piezoresistivity and temperature effects. Due to the parametric nature of the model, the influence of key parameters, such as particle size and tunneling distance, can expediently be explored. The capabilities of the modeling framework were demonstrated considering epoxy nanocomposites reinforced with silver particles. Model outputs were contrasted with available numerical and experimental results, and good qualitative agreement and acceptable quantitative agreement were ascertained. Reasons for quantitative differences are seen in the nanocomposite morphology created by the model, i.e., well-dispersed and homogenously distributed filler particles, which is in contrast with experimental works featuring materials with a typically clustered nanoparticle morphology. Future work will explore the effects of particle clustering, which can be implemented in the model by expanding the particle collision algorithm in the model generation step to not only avoid particle intersection but also enforce particle clustering. The analyses that were performed with the stochastic finite element analysis framework and presented in the present contribution demonstrate the advantages of the developed modeling approach in terms of versatility, time, and cost for exploring different materials systems as compared to experimental campaigns, analytical models, and other numerical techniques.

## Figures and Tables

**Table 1:** Electrical properties of the polymer matrix and filler particles [10].

	Epoxy Matrix	Nano-Silver Particles
Isotropic Electrical Resistivity [Ωm]	1.00 × 10^10^	1.59 × 10^-8^

**Table 2:** Mechanical properties of nano-silver particles and the polymer matrix.

	Epoxy Matrix	Nano-Silver Particles
Density [kg/m^3^]	1280	10,300
Modulus of Elasticity [GPa]	3.0	476
Poisson’s Ratio	0.4	0.36

**Table 3:** Thermal coefficient of expansion of fillers and the matrix.

	Epoxy Matrix	Nano-Silver Particles
Coefficient of Thermal Expansion [K^-1^]	45 × 10^−6^	18.0 × 10^−6^

**Table 4:** Effective electrical conductivity results and statistical analyses for a silver/epoxy nanocomposite with a particle size of 3 nm, tunneling distance of 1.5 nm, and 30 vol% filler loading.

Mean Value [S/m]	3.163 × 10^6^
Median Value [S/m]	3.239 × 10^6^
Standard Deviation [S/m]	0.218 × 10^6^
Variance [S/m]	1.03 ×10^11^
Skewness [/]	0.432
Kurtosis	−0.383
95% Confidence Interval [S/m]	0.090 × 10^6^
